# Novel *PPFIA1*-*ALK*, *ALK*-*C2orf91*(intergenic) double-fusion responded well to alectinib in an advanced lung adenocarcinoma patient: a case report

**DOI:** 10.3389/fonc.2023.1264820

**Published:** 2023-08-29

**Authors:** Lingxin Yan, Jiayu Zheng, Qingyun Pan, Yuxian Liang, Pengli Yu, Quanfang Chen

**Affiliations:** ^1^ Department of Pulmonary and Critical Care Medicine, The First Affiliated Hospital of Guangxi Medical University, Nanning, China; ^2^ Geneplus-Beijing Institute, Beijing, China

**Keywords:** PPFIA1-ALK, alectinib, NSCLC, NGS, non-reciprocal fusion

## Abstract

Non-small cell lung cancers (NSCLCs) with anaplastic lymphoma kinase (ALK)-rearrangement have favorable responses to ALK inhibitors. However, *ALK* fusion mutations harbored approximately 90 distinct fusion partners. Patients with different *ALK* fusions might respond distinctly to different-generation ALK inhibitors. In this case report, we identified a novel non-reciprocal *ALK* fusion, *ALK-C2orf91*(intergenic) (A19: intergenic) and *PPFIA1*-*ALK* (P2:A20), by next-generation DNA sequencing in an advanced lung adenocarcinoma patient. After 2 months of alectinib, the targeted lung lesion regressed significantly, and evaluation of therapeutic efficiency indicated partial response. To date, the patient had achieved 12 months of progression-free survival from alectinib treatment. Our study extended the spectrum of *ALK* fusion partners in *ALK*-positive NSCLC, and we reported a new *ALK* fusion, *PPFIA1*-*ALK* and *ALK*-*C2orf91*(intergenic), and its sensitivity to alectinib firstly in lung cancer. We believe that this case report has an important clinical reference.

## Introduction

*ALK* gene rearrangement is one of the most important driver mutations in non-small cell lung cancer (NSCLC) occurring in 3%–7% of the cases (1). Echinoderm microtubule-associated protein-like 4 (*EML4*)-*ALK* is the most common fusion variant, accounting for over 80% of fusion partners. With the increasing coverage of next-generation DNA sequencing, more than 90 rare *ALK* fusion subtypes have been discovered in NSCLC, such as kinesin family member 5B (*KIF5B*)-*ALK* and striatin gene (*STRN*)-*ALK* (2). First- to third-generation anaplastic lymphoma kinase (ALK) tyrosine kinase inhibitors (TKIs), including crizotinib, alectinib, and ceritinib, have brought outstanding efficacy and tolerability to patients (3–5). However, for the newly discovered fusion variants, especially those missing *EML4-ALK* fusion mutations, it is necessary to evaluate the clinical effectiveness of ALK-TKI in the first line or even the posterior line, and it is helpful to infer possible new therapeutic measures in the face of diverse rare fusion targets. Here, an advanced lung adenocarcinoma patient with *PPFIA1*-*ALK* and *ALK*-*C2orf91*(intergenic) double-fusion variants is sensitive to first-line alectinib.

## Case presentation

**Figure 1:**
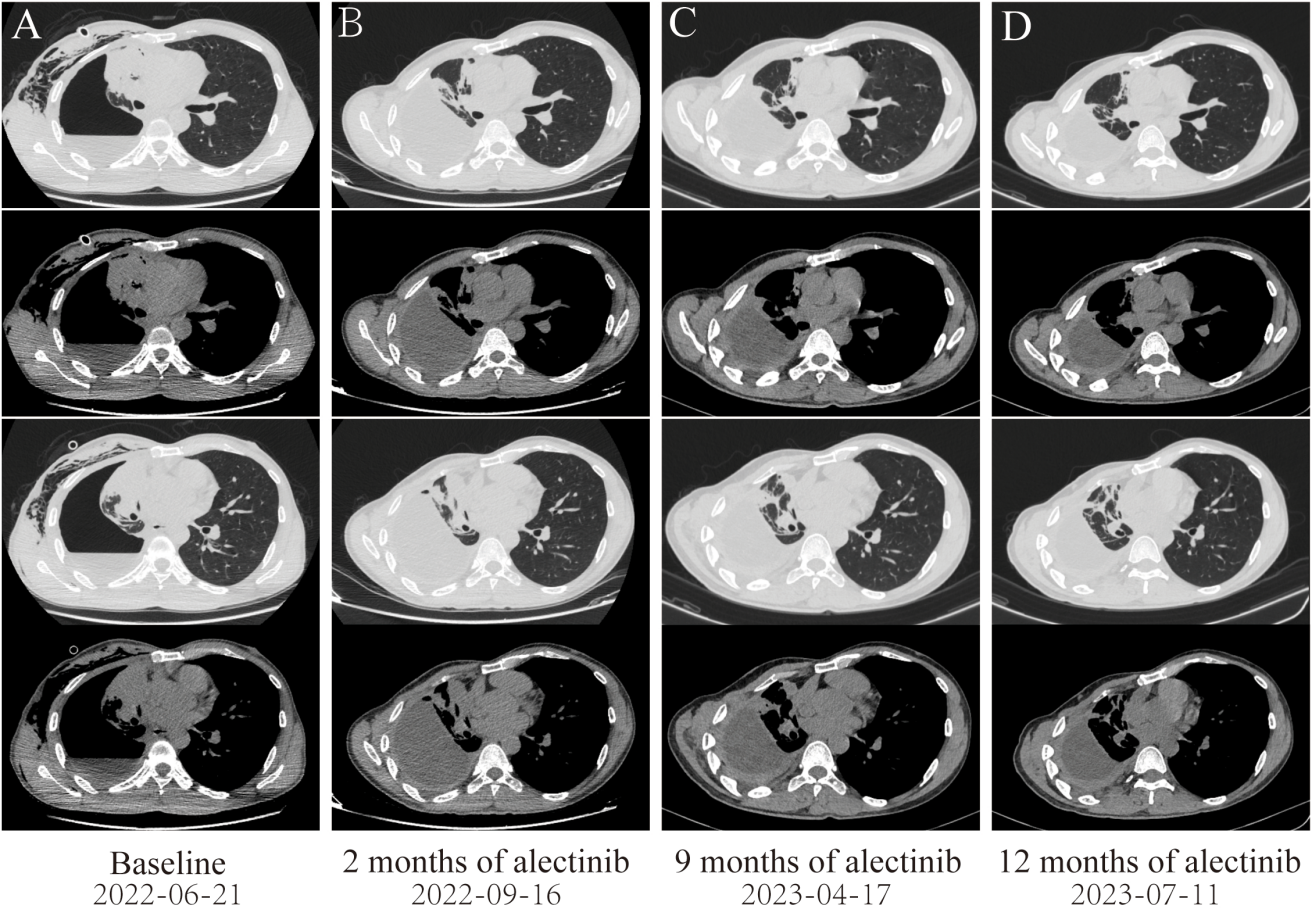
Dynamic imaging of lung lesions at different stages of treatment. **(A)** Lung lesions at diagnosis. **(B)** After targeted therapy with alectinib for 2 months, the right lung tumor was significantly reduced, resulting in partial response (PR). **(C)** After targeted therapy with alectinib for 9 months, the tumor in the right lung continued to shrink. **(D)** After targeted therapy with alectinib for 12 months, the right lung tumor tends to be stable.

**Figure 2:**
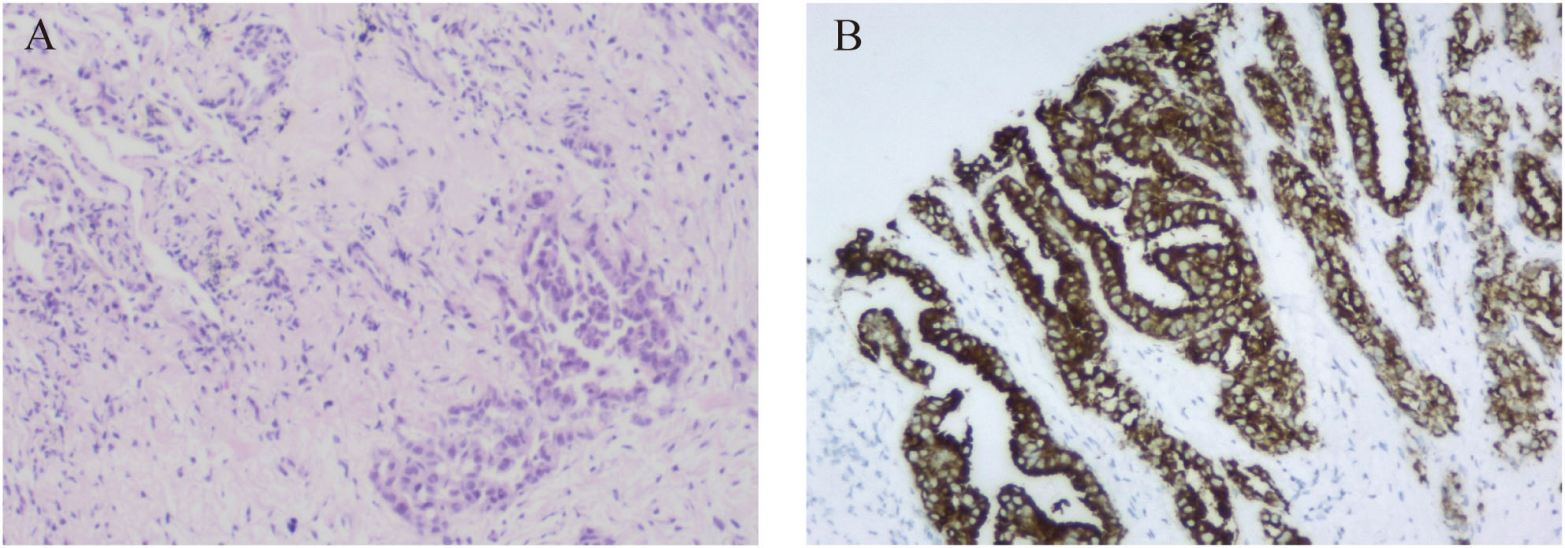
Pathological examination of the patient. **(A)** Lung tissue biopsy specimen (hematoxylin and eosin staining, magnification ×100). **(B)** The immunohistochemistry demonstrated positive expression for ALK (D5F3 Ventana).

**Figure 3:**
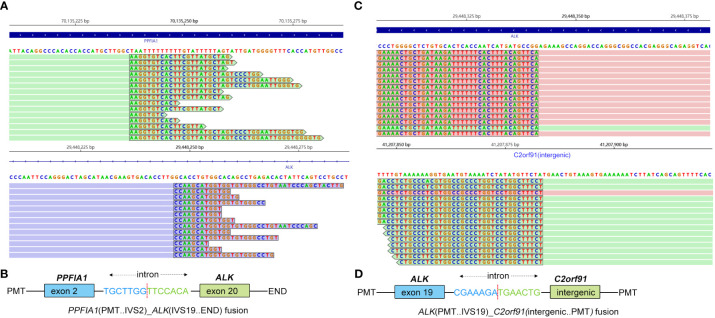
Identification of *PPFIA1*-*ALK* and *ALK*-*C2orf91*(intergenic) double-fusion variants by next-generation sequencing. **(A)** Sequencing reads of *PPFIA1*-*ALK* were visualized by the Integrative Genomics Viewer (IGV). **(B)** The schematic structure of the genomic DNA sequence shows the *PPFIA1*-*ALK* fusion points. **(C)** Sequencing reads of *ALK*-*C2orf91*(intergenic) were visualized by the IGV. **(D)** The schematic structure of the genomic DNA sequence shows the *ALK*-*C2orf91*(intergenic) fusion points.

A 46-year-old non-smoking Chinese man, without a family history of genetic disease, presented to another hospital in June 2022, with a cough for over 1 month and shortness of breath for approximately 10 days. A computed tomography (CT) scan revealed hydropneumothorax, compression atelectasis (approximately 80% collapse) and tumor mass of the right lung, and multiple nodules in the left lung (**Figure 1A**). He received pleural space drainage, and malignant cells were found in the pleural effusion. Then, he visited our hospital for further treatment. The pathologic results of ultrasound-guided percutaneous lung biopsy showed infiltrating growth of the allotypic glandular duct (**Figure 2A**), and the lesions tend to be pulmonary adenocarcinoma, with strongly positive expression for thyroid CK7, TTF-1, and Napsin A and negative for CK5/6, P40, and P60 as assayed by immunohistochemical staining. Bone scan, brain CT scan, and ultrasonography of the abdominal system, urinary system, and cardiovascular system identified no evidence of metastatic disease. Consequently, the patient was diagnosed with right lung adenocarcinoma with pleural metastasis (cT4N0M1a, stage IVa). To identify targetable mutations, the tumor biopsy specimen was sequenced by capture-based next-generation DNA sequencing with a panel containing 73 cancer-related genes ( (6) gene list is shown in **Supplementary Table 1**, Beijing Geneplus Technology Co., Ltd.). The mean effective depth of coverage of the sequence was 2,032×. Non-reciprocal ALK fusion *PPFIA1-ALK* (P2:A20) (abundance, 10%) and *ALK-C2orf91* (A19, intergenic) (abundance, 13%) was identified in tissue (**Figure 3**). No other gene mutation was detected. Immunohistochemistry staining revealed that the tumor had positive expression for ALK with the Ventana D5F3 (**Figure 2B**). Therefore, based on guidelines and clinical studies, combined with the patient’s wishes, he received alectinib (600 mg twice daily) as first-line therapy on July 15, 2022.

After 2 months of alectinib therapy, a follow-up CT scan found the right lung tumor remarkably reduced, thus achieving a partial response based on Response Evaluation Criteria in Solid Tumors [RECIST] version 1.1 (**Figure 1B**). Follow-up CT scans at 9 months and 12 months showed reduced stable disease (**Figures 1C, D**). At the cutoff date of this study, the patient had been receiving alectinib for 12 months with no complaints of discomfort and no adverse effects.

## Discussion

In summary, this is the first case to describe a novel *PPFIA1-ALK* and *ALK-C2orf91* (intergenic) double-fusion lung adenocarcinoma patient who is sensitive to alectinib. Multiple *ALK* fusion types have been reported in NSCLC patients, among which different fusion partners and different breakpoint variants may affect the response to ALK-TKIs (7, 8). Previous reports described that *HIP1-ALK* (H21:A20) and *HIP1-ALK* (H30:A20) responded well to crizotinib (9, 10), while *HIP1-ALK* (H19:A20) showed resistance to crizotinib with progression of pleural effusion (8). Therefore, determining the efficacy of different *ALK* fusions to different ALK inhibitors is vital for personalized therapeutic decision-making.

As far as we know, *PPFIA1*-*ALK* fusion has not been reported in lung cancer before. *PPFIA1* is a putative invasion suppressor gene located in the 11q13 region, while *ALK* is located in the short arm of chromosome 2. Amplification and overexpression of *PPFIA1* were reported to be associated with poor prognosis in breast cancer (11). In the current case, exon 2 of the *PPFIA1* gene rearranged with exon 20 of the *ALK* gene to form a new fusion gene, *PPFIA1*-*ALK*. Although there is no direct evidence to support *PPFIA1*-*ALK* as a driver mutation, considering that *PPFIA1* has been reported to be highly expressed in lung tissues (12), there is a possibility that *PPFIA1*‐*ALK* rearrangement is a driver mutation.

According to the global ALEX study, alectinib showed superior progression-free survival (PFS) versus crizotinib in untreated *EML4-ALK* fusion NSCLC, irrespective of the *EML4-ALK* variant (13). For patients with 3′-*ALK* fusion, the retention of 5′-*ALK* fusion was defined as non-reciprocal fusion, which was predictive for worse PFS at first-line crizotinib (14). Therefore, *ALK*-*C2orf91* (A19: intergenic) as 5′-ALK and *PPFIA1*-*ALK* (P2:A20) as 3′-ALK in our case were defined together as a non-reciprocal *ALK* fusion. Zeng reported an NSCLC patient with non-reciprocal *ALK* fusion after resistance to first-line gefitinib who responded to alectinib and had a PFS of more than 26 months. Similar to the previous report, the patient in the present case was also sensitive to alectinib and had been receiving alectinib for 12 months.

## Conclusions

In summary, this case presented a rare novel non-reciprocal *PPFIA1-ALK* and *ALK-C2orf91* (intergenic) double-fusion mutation sensitive to first-line alectinib.

## Data availability statement

The original contributions presented in the study are included in the article/**Supplementary material**. Further inquiries can be directed to the corresponding author.

## Ethics statement

The studies involving humans were approved by Ethics Committee of First Affiliated Hospital of Guangxi Medical University. The studies were conducted in accordance with the local legislation and institutional requirements. The participants provided their written informed consent to participate in this study. Written informed consent was obtained from the individual(s) for the publication of any potentially identifiable images or data included in this article.

## Author contributions

LY: Data curation, Writing – original draft, Writing – review & editing. JZ: Data curation, Writing – review & editing. QP: Data curation, Writing – review & editing. YL: Data curation, Writing – review & editing. PY: Investigation, Writing – original draft, Writing – review & editing. QC: Funding acquisition, Supervision, Writing – review & editing.
